# Reorganization of supramammillary–hippocampal pathways in the rat pilocarpine model of temporal lobe epilepsy: evidence for axon terminal sprouting

**DOI:** 10.1007/s00429-014-0800-2

**Published:** 2014-06-03

**Authors:** Rabia Soussi, Jean-Luc Boulland, Emilie Bassot, Hélène Bras, Patrice Coulon, Farrukh Abbas Chaudhry, Jon Storm-Mathisen, Lotfi Ferhat, Monique Esclapez

**Affiliations:** 1 INSERM, UMR 1106, Institut de Neurosciences des Systèmes - INS, 13385 Marseille, France; 2 Aix-Marseille University, 13385 Marseille, France; 3 Biotechnology Center, Institute of Basic Medical Sciences and Center for Molecular Biology and Neuroscience, University of Oslo, 0317 Oslo, Norway; 4 Department of Anatomy, Institute of Basic Medical Sciences and Center for Molecular Biology and Neuroscience, University of Oslo, 0317 Oslo, Norway; 5 CNRS, UMR 7289, Institut de Neurosciences de la Timone, 13385 Marseille, France; 6 CNRS, UMR 7259, NICN, 13385 Marseille, France

**Keywords:** Hippocampus, Dentate gyrus, GAD, VGAT, VGLUT2, Hypothalamus, SuM

## Abstract

In mesial temporal lobe epilepsy (MTLE), spontaneous seizures likely originate from a multi-structural epileptogenic zone, including several regions of the limbic system connected to the hippocampal formation. In this study, we investigate the structural connectivity between the supramammillary nucleus (SuM) and the dentate gyrus (DG) in the model of MTLE induced by pilocarpine in the rat. This hypothalamic nucleus, which provides major extracortical projections to the hippocampal formation, plays a key role in the regulation of several hippocampus-dependent activities, including theta rhythms, memory function and emotional behavior, such as stress and anxiety, functions that are known to be altered in MTLE. Our findings demonstrate a marked reorganization of DG afferents originating from the SuM in pilocarpine-treated rats. This reorganization, which starts during the latent period, is massive when animals become epileptic and continue to evolve during epilepsy. It is characterized by an aberrant distribution and an increased number of axon terminals from neurons of both lateral and medial regions of the SuM, invading the entire inner molecular layer of the DG. This reorganization, which reflects an axon terminal sprouting from SuM neurons, could contribute to trigger spontaneous seizures within an altered hippocampal intrinsic circuitry.

## Introduction

Mesial temporal lobe epilepsies (MTLEs) are among the most common forms of drug-resistant partial epilepsy in adults (Engel 1996). These epilepsies are often associated with an antecedent brain insult, such as prolonged febrile seizures, status epilepticus, brain trauma or meningitis, and with hippocampal sclerosis (HS) (Wieser 2004). These observations have led to the concept that MTLE results from an aberrant hippocampal network triggered by an initial brain insult. However, many electro-clinical studies provide evidence that the abnormal network responsible for seizure generation, the epileptogenic zone, cannot be restricted to the hippocampal formation. In MTLE, seizures originate from a multi-structural epileptogenic zone, which involves several limbic structures of the temporal lobe including the hippocampus, the amygdala and the entorhinal cortex (For review see Kahane and Bartolomei 2010).

Animal models of MTLE including the pilocarpine model of chronic limbic seizures demonstrate that an initial brain insult caused by status epilepticus leads to a complex time-dependent reorganization of the intrinsic gamma-aminobutyric acid (GABA)-ergic (Obenaus et al. 1993; Houser and Esclapez 1996; Esclapez et al. 1997; Esclapez and Houser 1999; Cossart et al. 2001; Dinocourt et al. 2003; Kobayashi and Buckmaster 2003; Sloviter et al. 2003; Peng et al. 2013) and glutamatergic (Mello et al. 1993; Okazaki et al. 1995; Esclapez et al. 1999; Lehmann et al. 2001; Okazaki and Nadler 2001; Buckmaster et al. 2002; Dashtipour et al. 2003; Sloviter et al. 2003; Boulland et al. 2007; Jiao and Nadler 2007; Ribak et al. 2012) hippocampal networks. These complex reorganizations of the hippocampal intrinsic neuronal networks lead to a marked homeostatic unbalance between excitation and inhibition not only during the chronic stage (Cossart et al. 2001; El-Hassar et al. 2007) but also during the latent period when the animals do not yet display spontaneous seizures (Kobayashi and Buckmaster 2003; El-Hassar et al. 2007). Furthermore, the ratio of the glutamatergic drive to the overall synaptic drive received by CA1 pyramidal cells during the latent period is not further modified in epileptic animals (El-Hassar et al. 2007). This suggests that the reorganization of hippocampal intrinsic networks is not sufficient by itself to generate seizures. Therefore, in this animal model, the epileptogenic zone likely involves broader reorganizations within and between different brain regions of the limbic system.

In this study, we examined a potential reorganization of the connectivity between the hypothalamus and the hippocampal formation, more specifically the hippocampal afferents from the supramammillary region (SuM), which is the main hypothalamic structure innervating the hippocampal formation. This hypothalamic region is involved in the regulation of hippocampal theta rhythms (Kirk and McNaughton 1991, 1993; Kocsis and Vertes 1994; Vertes and Kocsis 1997; Kocsis and Kaminski 2006) and therefore plays a crucial role in the control of several hippocampus-dependent cognitive functions but also emotional behavior, including stress and anxiety (Pasquier and Reinoso-Suarez 1976; Richmond et al. 1999; Pan and McNaughton 2002; Santin et al. 2003; Pan and McNaughton 2004; Shahidi et al. 2004). Interestingly, it is known that hippocampal theta rhythms and hippocampus-dependent memory functions are altered in the pilocarpine model of MTLE (Chauvière et al. 2009; Marcelin et al. 2009) as well as in human MTLE (Bettus et al. 2008) and that in these epilepsies, the occurrence of seizures is particularly sensitive to stress (Lanteaume et al. 2009).

Although studies in human MTLE (Maglóczky et al. 2000) and a related animal model (Skyers et al. 2003) have suggested axonal sprouting of SuM neurons as one possible explanation for an increase in calretinin- and μ-opioid receptor-labeled terminals in the dentate gyrus, respectively, such actual growth and reorganization of SuM–hippocampal pathways remain to be demonstrated. The current study was designed to address this issue as well as to determine the time course of these potential structural reorganizations at the different stages of development of the pilocarpine-induced epilepsy (latent and chronic stages). For this purpose, we performed anterograde-tracing experiments in combination with immunohistochemical experiments for the vesicular glutamate transporter 2 (VGLUT2, Fremeau et al. 2001; Herzog et al. 2001), the synthesizing enzyme for GABA, glutamic acid decarboxylase 65 (GAD65, Esclapez et al. 1993, 1994) and the vesicular GABA transporter (VGAT, McIntire et al. 1997; Chaudhry et al. 1998), to discriminate fibers and axon terminals, originating from the different SuM regions. Indeed in naïve rat, the distinct SuM–hippocampal pathways can be differentiated, based on their anatomical and neurochemical features. The first pathway originates from neurons in the lateral region of the SuM (SuML) and innervates the supragranular layer of the dorsal dentate gyrus (DG) and, to a much lesser extent, the ventral DG (Haglund et al. 1984; Maglóczky et al. 1994; Vertes and McKenna 2000). This pathway displays a unique glutamatergic and GABAergic dual neurotransmitter phenotype. Its axon terminals co-express VGLUT2, GAD65 and VGAT (Soussi et al. 2010). The second pathway originates from neurons in the most posterior and medial part of the SuM (SuMM) and innervates the inner molecular layer of the ventral DG exclusively (Vertes 1992; Maglóczky et al. 1994) as well as the CA2/CA3a pyramidal cell layer (Maglóczky et al. 1994). The axon terminals from the SuMM contain VGLUT2 only (Soussi et al. 2010). To ascertain the neuronal source and targets of observed aberrant connectivity in pilocarpine-treated animals, we performed additional experiments including in situ hybridization for VGLUT2 mRNA as well as retrograde-tracing methods with rabies virus and immunohistochemical labeling for the neuron-specific nuclear protein (NeuN).

## Materials and methods

Animal care and experimental procedures were performed in accordance with the European Community’s Council Directive 86/609/EEC and were approved by Aix-Marseille University Chancellor’s Animal Research Committee.

### Animals and pilocarpine treatment

Young adult male Wistar rats (180–200 g; Charles River, France) were injected with a low dose of methyl scopolamine nitrate (1 mg/kg, i.p.; Sigma, St Louis, MO), in order to minimize the peripheral effects of the pilocarpine hydrochloride (340 mg/kg; i.p.; Sigma) provided 30 min later to induce status epilepticus (SE). The period of SE was alleviated after 2 h with diazepam (8 mg/kg, i.p.; Roche, Boulogne-Billancourt, France). Pilocarpine-treated animals (*n* = 20) were observed periodically for general behavior and occurrence of spontaneous seizures. These animals were studied at several post-SE intervals: at 1 and 2 weeks, when animals displayed an apparently normal behavior (*n* = 3 at each interval), and at 2, 4 and 12 months, when the animals have developed spontaneous recurrent seizures (*n* = 5, 6 and 3, respectively). We previously demonstrated, using the same experimental conditions and continuous in vivo electroencephalographic recordings, that in these pilocarpine-treated rats, the first spontaneous seizures occur during the second or third week (range 12 and 18th day) after SE (Marcelin et al. 2009). Each group of pilocarpine-treated animals (1, 2 weeks, 2, 4 and 12 months) was compared to a group of age-matched untreated rats (controls; *n* = 15).

### Anterograde tracer injections

The anterograde tracer, Biotin-Dextran-Amine (BDA 2 %; MW 3,000; Invitrogen, Carlsbad, CA), was injected under stereotaxic condition into the SuML and SuMM according to previously described protocol (Soussi et al. 2010). Control (*n* = 6) and pilocarpine-treated rats at 2 and 4 months (*n* = 6) were anesthetized with chloral hydrate (400 mg/kg; i.p.; Fisher Scientific, Loughborough, UK), secured in a stereotaxic frame (David Kopf, Tujunga, CA) and pressure-injected with 300 nL (30 nL/min during 10 min) of BDA either into the SuML at the following coordinates: 3.9–4 mm posterior to bregma (AP), 0.5–0.7 mm lateral to the midline (L) and 8.9–9 mm ventral to the cortical surface (V) or into the SuMM at the following coordinates: AP = 4.3–4.7 mm *L* = 0.2 mm and *V* = 8.8 mm. After the completion of injection procedures, the syringe was removed and the skin sutured. The animals were treated with topical anesthetic and then returned to their home cages for a survival period of 8–10 days to allow proper anterograde transport of the tracer.

### Rabies virus retrograde tracer

Rabies virus (RV) retrograde tracings were performed in order to obtain a Golgi-like labeling (Salin et al. 2008) of DG granule cells. Vaccinated personnel conducted all experiments at the appropriate biosafety containment level. The strain of RV used in this study was the Challenge Virus Standard (CVS) (Bras et al. 2008; Ugolini 2010; Coulon et al. 2011). The virus stock as cell culture supernatant in minimal essential medium (4.10^7^ plaque-forming units/mL) was kept frozen at −80 °C until use.

Under chloral hydrate general anesthesia, pilocarpine-treated rats (*n* = 2, at 2 months) were pressure-injected under stereotaxic condition within the stratum lucidum of the CA3b region in the dorsal hippocampus with RV at a 200 nL final volume. After completion of the injection procedures, the syringe was removed and the skin was sutured. The animals were treated with local anesthetic, returned to their cages kept at the appropriate biosafety containment level for a survival period of 42 h to observe an optimal RV retrograde labeling of the dendritic arbor of dentate granule cells.

### Tissue preparation

Rats were deeply anesthetized with choral hydrate (500 mg/kg; Fisher Scientific) and transcardially perfused with 4 % paraformaldehyde in 0.12 M sodium phosphate buffer, pH 7.4 (PB). The brains were removed from the skull, post-fixed for 1 h at room temperature (RT), rinsed in PB, cryoprotected in 20 % sucrose overnight, frozen on dry ice and sectioned coronally at 40 µm with a cryostat. Every tenth section was stained with cresyl violet in order to determine the general histological characteristics of the tissue within the rostro-caudal extent of the brain. From each rat, selected sections were processed for (1) immunohistochemical labeling for VGLUT2; (2) detection of tracers; (3) multiple labeling for detection of the anterograde or retrograde tracers and immunohistochemical detection of VGAT or GAD65 and VGLUT2; (4) double labeling for detection of NeuN and VGLUT2; and (5) nonradioactive in situ hybridization using VGLUT2 cRNA as a probe. Sections from control and pilocarpine-treated animals were always processed in parallel.

### Detection of the BDA anterograde tracer

The BDA-containing fibers and axon terminals were visualized using the avidin–biotin–peroxidase method as previously described (Soussi et al. 2010). Free-floating sections were pre-treated in 1 % H_2_O_2_ for 30 min, rinsed several time in 0.02 M potassium phosphate-buffered saline (KPBS, pH 7.2–7.4), then incubated overnight at RT in an avidin–biotinylated–peroxidase complex (Vectastain Elite ABC, Vector laboratories, Burlingame, CA) diluted 1:100 in KPBS containing 0.3 % Triton X-100 (Sigma). The following day, after several rinses in KPBS, sections from control and pilocarpine-treated animals were processed for 15 min in 3,3′-diaminobenzidine-HCl (DAB Sigma fast tablets; Sigma). Sections were subsequently mounted onto superfrost plus slides (Thermo scientific, Brunswick, Germany), dehydrated and coverslipped with Permount (Electron Microscopy Sciences, Washington, PA).

### Immunohistochemistry

#### Single immunohistochemical labeling for VGLUT2

Free-floating sections were processed for immunohistochemistry according to previously described protocol (Esclapez et al. 1994). Sections were pre-treated for 30 min in 1 % H_2_O_2_, rinsed in PB and KPBS, pre-incubated for 1 h in 3 % normal goat serum (NGS, Vector Laboratories) diluted in KPBS containing 0.3 % Triton X-100 and incubated overnight at RT in VGLUT2 rabbit polyclonal antiserum (1:5,000; Synaptic Systems, SYSY, Gottingen, Germany) diluted in KPBS containing 1 % NGS and 0.3 % Triton X-100. After several rinses in KPBS, sections were incubated for 1 h at RT in biotinylated goat anti-rabbit immunoglobulin G (IgG; Vector Laboratories) diluted 1:200 in KPBS containing 3 % NGS and then for 1 h at RT in Vectastain Elite ABC diluted 1:100 in KPBS. Sections from control and pilocarpine-treated rats were processed for the same period of time (15 min) in DAB, rinsed in KPBS, mounted onto Superfrost Plus slides, dehydrated and coverslipped with Permount.

#### Simultaneous immunohistochemical detection for NeuN and VGLUT2

Sections were pre-treated in 1 % H_2_O_2_ for 30 min, rinsed several times in PB and KPBS and pre-incubated for 1 h in KPBS containing 1 % blocking reagent (BR, Bœrhinger Manheim, Germany) and 0.3 % Triton X-100. Then, they were incubated overnight in a mixture of VGLUT2 rabbit antiserum (1:5,000) and NeuN mouse monoclonal antibody (1:4,000; Millipore, Temecula, CA) diluted in KPBS containing 0.5 % BR and 0.3 % Triton X-100. The following day, sections were first processed for detection of VGLUT2 as described above. After extensive rinses in KPBS, sections were processed for detection of NeuN. They were incubated for 1 h in biotinylated horse anti-mouse IgG (1:200; Vector laboratories) diluted in KPBS containing 0.5 % BR, rinsed in KPBS, then incubated for 1 h in avidin–biotin–alkaline phosphatase complex diluted in KPBS, and finally revealed for 10 min in Fast Red (Fast Red tablets, Sigma) diluted in 0.1 M Tris–HCl buffer (TB, pH 8.2). Sections were mounted onto Superfrost slides and coverslipped with Crystal Mount (Biomeda, Foster City, CA).

#### Multiple immunofluorescence labeling for BDA or Rabies virus, VGLUT2 and GAD65 or VGAT

Free-floating sections were processed for multiple immunofluorescence as previously described (Soussi et al. 2010). Briefly, they were rinsed in KPBS, then incubated for 1 h at RT in 3 % normal donkey serum (NDS, Jackson ImmunoResearch Laboratories, Suffolk, UK) and diluted in KPBS containing 0.3 % Triton X-100. For simultaneous detection of BDA, VGLUT2 and VGAT or GAD65, sections were incubated overnight at RT in a solution containing goat anti-biotin (1:200; Vector Laboratories), guinea pig anti-VGLUT2 (1:5,000; Millipore) and rabbit anti-VGAT (1:2,000; SYSY) or mouse anti-GAD65 (1:100; MAB 351R, Millipore) diluted in KPBS containing 0.3 % Triton X-100 and 1 % NDS. After several rinses in KPBS, they were incubated for 2 h in Alexa 488-conjugated donkey anti-goat IgG (1:100; Invitrogen, Burlingame, CA), Cy5-conjugated donkey anti-guinea pig IgG (1:100; Jackson ImmunoResearch Laboratories) and Cy3-conjugated donkey anti-rabbit IgG or anti-mouse IgG (1:100; Jackson ImmunoResearch Laboratories) diluted in KPBS. For simultaneous detection of RV, VGLUT2 and VGAT, sections were incubated overnight at RT in a solution containing mouse anti-RV (1:5,000; Raux et al. 1997), guinea pig anti-VGLUT2 (1:5,000) and rabbit anti-VGAT (1:2,000) diluted in KPBS containing 0.3 % Triton X-100 and 1 % NDS and then for 2 h in Alexa 488-conjugated donkey anti-mouse IgG (1:100), Cy5-conjugated donkey anti-guinea pig IgG (1:100, Jackson ImmunoResearch Laboratories) and Cy3-conjugated donkey anti-rabbit IgG. All sections were mounted on superfrost-coated slides, dried and coverslipped with Gel/Mount (Biomeda).

#### Controls

In all cases, no cross-reactivity was detected when one or two of the primary antibodies were omitted.

Sections were analyzed either with a Leica TCS SP (Leica, Wetzlar Germany) or a Zeiss LSM 5 Pascal (Zeiss, Jena, Germany) laser-scanning microscopes using sequential acquisition of separate wavelength channels to avoid fluorescence cross talk. Single confocal optical slices and z-stacks of 10–25 confocal optical slices (2,048 × 2,048 pixels) spaced 200–300 nm were acquired with a 100X objective (oil immersion, 1.4 numerical aperture), a numerical zoom 2 (37.8 nm/pixel) or zoom 8 (9.4 nm/pixel) and with the pinhole set at 1 Airy unit. Images were displayed with the NIH ImageJ software (Abràmoff et al. 2004).

### Quantification of VGLUT2 and VGLUT2–VGAT immunolabeling

Quantification of bouton densities from BDA-tracing experiments does not allow comparative analysis between animals. Indeed, the number of BDA-labeled fibers and boutons may vary for each animal depending on tracer uptake efficiency by the neurons (amount of labeled cells). Therefore, in the DG, the densities of boutons, originating from SuML and SuMM neurons, were estimated by the densities of axon terminals displaying SuML and SuMM neurochemical phenotype, i.e., labeled for VGLUT2/VGAT and VGLUT2 only, respectively. The densities of VGLUT2/VGAT- and VGLUT2 only labeled terminals were assessed by the quantification of immunolabeling for VGLUT2/VGAT and VGLUT2 only, respectively. These analyses were performed on sections from both control (*n* = 3) and 4-month pilocarpine-treated (*n* = 3) rats. Single optical confocal images were acquired with Zeiss LSM 5 Pascal laser-scanning microscope and analyzed with the software provided by the microscope manufacturer (LSM 5 Examiner, Zeiss). All images were acquired from the suprapyramidal blade of the dorsal and ventral DG, using identical parameters for control and pilocarpine-treated animals. The densities of labeling for VGLUT2 only and for VGLUT2–VGAT were estimated by counting the number of positive pixels in each dentate gyrus (dorsal and ventral) and in two regions of interests (ROIs): (1) the granule cell layer (GCL) including the narrow zone superficial to the granule cells defined as the supragranular layer (SGL) and (2) the inner one-third of the molecular layer (IML) as illustrated in Fig. 5b. For each channel, an identical bottom threshold was applied throughout the analyses, and only the pixels with a value above this threshold were counted. When a pixel had a value above the threshold in both channels, it was counted as double positive. The number of counted pixels was reported to the area of the ROI so that the variable shapes of the ROIs drawn were not a source of bias. The average numbers of pixels representing the average labeling densities for VGLUT2 only and VGLUT2–VGAT (±SEM) were calculated in each ROI for each group of control and 4-month pilocarpine-treated rats. The data were analyzed statistically with a mixed model analysis of variance (ANOVA) and Student’s *t* test.

### In situ hybridization

#### Probe synthesis

The VGLUT2 probes used in this study were digoxigenin-labeled riboprobes obtained by in vitro transcription of a rat VGLUT2 cDNA (Gift from Dr S. El Mestikawy). This cDNA (539 bp) was inserted into the pCR-TOPO-II vector (Invitrogen) for in vitro transcription. The transcription was carried out with the nonradioactive RNA labeling kit (Roche Diagnostics, Meylan, France). The recombinant plasmid containing the VGLUT2 cDNA insert was linearized with Xho I and transcribed with Sp6 RNA polymerase to obtain the antisense probe or linearized with Hind III and transcribed with T7 to obtain the sense probe. The labeling efficiency of the digoxigenin-labeled probes for VGLUT2 mRNA was determined each time by direct immunological detection on dot blots with a nucleic acid detection kit (Roche Diagnostics). The intensity of the signal for each probe was compared with a serial dilution of digoxigenin-labeled control RNA of known concentration. Only antisense and sense VGLUT2 probes with comparable signal intensity (comparable labeling efficiency), as determined in dot blots, were used for in situ hybridization.

#### Hybridization and detection

Free-floating sections from control (*n* = 3) and 2- to 4-month pilocarpine-treated (*n* = 6) rats were processed for VGLUT2 in situ hybridization according to a previously described protocol (Esclapez et al. 1993; Boulland et al. 2007). Briefly, after several pre-treatments to enhance penetration of the probes, sections were incubated for 1 h at RT in the pre-hybridization solution, and then overnight at 50 °C in the hybridization solution, consisting of the pre-hybridization solution with the addition of 0.2 ng/µl digoxigenin-labeled RNA probe, 100 mM dithiothreitol (DTT) and 4 % dextran sulfate. After hybridization, sections were treated with ribonuclease A (50 µg/mL) for 30 min at 37 °C following by low- to high-stringency washes performed with decreasing concentrations of saline sodium citrate solution (SSC), ending with an incubation in 0.1× SSC, 10 mM sodium thiosulfate for 30 min at 55 °C. Sections were then processed for immunodetection of the digoxigenin label by means of a nucleic acid detection kit (Roche Diagnostic). They were incubated overnight at 4 °C in alkaline phosphatase-conjugated sheep antibodies to digoxigenin diluted 1:1,000, then in a chromogen solution containing nitroblue tetrazolium (NBT) and 5-bromo-4-chloro-3-indolyl phosphate (BCIP) reagents. The times in the chromogen solution were determined according to previously described protocols (Esclapez and Houser 1999; Boulland et al. 2007). Sections from control and pilocarpine-treated animals were incubated in the chromogen solution until optimal staining was achieved for VGLUT2 mRNA in each animal group (control and epileptic pilocarpine-treated rats). For VGLUT2 probe, the optimal color reaction times (7.5 h) were similar for all sections from all animal groups (control and pilocarpine-treated animals). In all experiments, the color reaction was stopped by rinsing the sections in TB, pH 8.0 containing 1 mM EDTA. Sections were then mounted on gelatin-coated slides, dried and coverslipped in an aqueous mounting medium (Crystal/Mount, Biomeda).

## Results

### Changes in VGLUT2 immunolabeling in the dentate gyrus of pilocarpine-treated animals

**Fig. 1:**
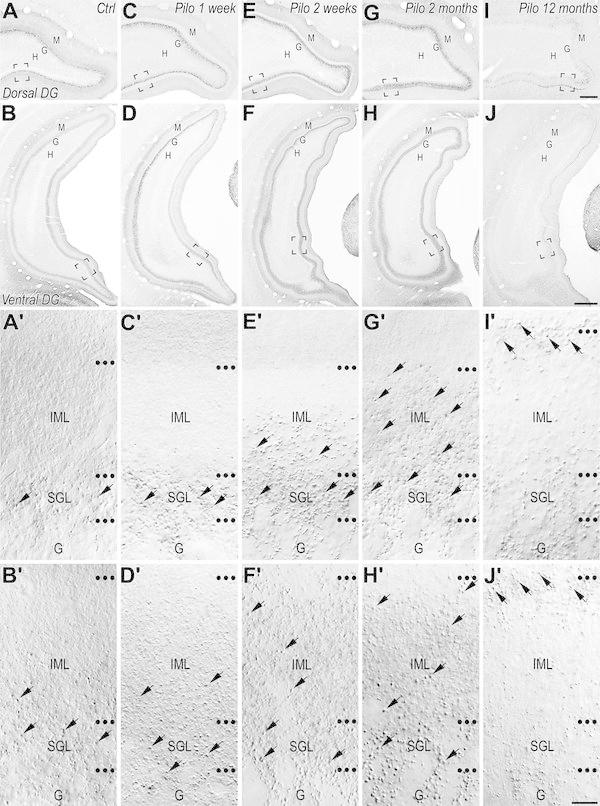
Comparison of immunohistochemical labeling for VGLUT2 in coronal sections through the rostro-caudal extent of the dentate gyrus from control (**a**, **b**′) and pilocarpine-treated animals at 1 week (**c**, **d**′), 2 weeks (**e**, **f**′), 2 months (**g**, **h**′) and 12 months (**i**, **j**′). **a**′–**j**′ panels correspond to high magnification of the region outline in panels **a**–**j**, respectively. **a**–**b**′ In a control rat, VGLUT2 immunolabeling was present in the granular (G) and molecular (M) layers of the dentate gyrus (DG) at rostral (**a**) and caudal (**b**) levels. Virtually no labeling was observed in the hilus (H). At high magnification (**a**′, **b**′), VGLUT2 labeling included both punctate structures (representative examples pointed by *arrows*) and a diffuse labeling. The punctate structures, presumed axon terminals from the supramammillary nucleus neurons, displayed different patterns of distribution along the dorsal to ventral axis of the dentate gyrus (**a**, **b**). These terminals were concentrated in the supragranular layer (SGL) of the dorsal region of DG (**a**′) and were much sparser distributed throughout the inner molecular layer (IML) in the ventral DG (**b**′). The diffuse immunolabeling for VGLUT2 was observed in the inner and outer one-third of the molecular layer (**a**, **b**). **c**–**c**′ In a pilocarpine-treated rat at 1 week, a decrease of the diffuse immunolabeling for VGLUT2 was evident in the IML of the dorsal (**c**, **c**′) but not in the ventral (**d**, **d**′) DG. As in control (**a**′, **b**′), many VGLUT2-containing terminals (*arrows*) were present in the SGL of the dorsal (**c**′) and ventral DG (**d**′). **e**–**f**′ In a pilocarpine-treated rat at 2 weeks, the loss of VGLUT2 diffuse labeling was still evident in the IML of the dorsal DG (**e**, **e**′). In addition to the numerous VGLUT2-containing terminals (*arrows*) observed in the SGL, many of them were also present in the IML in the dorsal DG (*arrows*; **e**′) and ventral IML (arrows; **f**′). **g**–**h**′ In an epileptic pilocarpine-treated rat at 2 months, numerous VGLUT2-containing terminals (*arrows*) were present in the entire IML throughout the rostro-caudal (**g**, **h**′) extent of the DG. An apparent recovery of diffuse labeling was observed in IML of the dorsal DG (**g**′). **i**–**j**′ In an epileptic animal at 12 months, VGLUT2 immunolabeling was clearly different from that observed in control and pilocarpine-treated rats at 1 and 2 weeks but also from epileptic animals at 2 months. VGLUT2-containing terminals displayed a double-band distribution pattern, these terminals being located in the SGL and in the uppermost part of the IML (*arrows*). A marked loss of the diffuse immunolabeling for VGLUT2 was observed now throughout the entire rostro-caudal level in the IML including in the ventral dentate gyrus. *H* hilus, *G* granule cell layer, *M* molecular layer, *IML* inner molecular layer, *Ctrl* control, *Pilo 1* *week* pilocarpine-treated animal at 1 week after SE, *Pilo 2* *weeks* pilocarpine-treated animal at 2 weeks after SE, *Pilo 2* *months* pilocarpine-treated animal at 2 months after SE, *Pilo 12* *months* pilocarpine-treated animal at 12 months after SE. *Scale bars* 200 µm in **a**, **c**, **e**, **g**, **i**; 500 µm in **b**, **d**, **f**, **h**, **j** and 10 µm in **a**′–**j**′

The pattern of VGLUT2 immunohistochemical labeling in the rat hippocampus has been reported previously (Fremeau et al. 2001; Herzog et al. 2001; Kaneko et al. 2002; Halasy et al. 2004; Boulland et al. 2009; Soussi et al. 2010). Our study focused on the dentate gyrus (DG) where the main differences were observed between control and pilocarpine-treated rats. A detailed description of VGLUT2 immunolabeling through the entire rostro-caudal extent of the DG was provided in control rats to compare to that of pilocarpine-treated rats. All control animals, regardless of their age, displayed the same patterns of immunolabeling for VGLUT2. In these control animals, the VGLUT2 immunolabeling showed both laminar- and regional-specific patterns within the DG (Fig. 1a–b′). At low magnification, whereas moderate to strong VGLUT2 immunolabeling were evident in the molecular and granule cell layers, very low levels or virtually no labeling were observed in the hilus and CA3c pyramidal cell layer, respectively, through the entire rostral (Fig. 1a) to caudal (Fig. 1b) extent of the DG. At higher magnification (Fig. 1a′, b′), the immunohistochemical labeling for VGLUT2 was characterized by large punctate structures (arrows) and by a thin diffuse labeling, as previously described (Fremeau et al. 2001; Kaneko et al. 2002; Halasy et al. 2004). The large punctate structures, observed through the entire rostro-caudal extent of the hippocampus displayed, however, different patterns of distribution along the dorsal to ventral axis of the DG. Whereas in the dorsal DG, these large punctate structures labeled for VGLUT2 were highly concentrated in the supragranular layer (SGL) delineating this narrow region superficial to the granule cells from the adjacent IML (Fig. 1a′, arrows), in the ventral DG, they were dispersed within SGL and adjacent IML (Fig. 1b′, arrows). These VGLUT2-labeled punctate structures correspond mainly to axon terminals from neurons located in the SuM (Boulland et al. 2009; Soussi et al. 2010). The diffuse VGLUT2 immunolabeling, observed in the DG, displayed a similar pattern of distribution along the rostro-caudal and dorso-ventral axes of the hippocampus with higher levels of labeling in the inner and outer one-third of the molecular layer (Fig. 1a, b). These diffuse labeling in the inner and outer one-third of the DG molecular layer has been suggested to correspond to axon terminals originating from hilar mossy cells and entorhinal cortex layer II/III neurons, respectively (Halasy et al. 2004).

The pattern of VGLUT2 immunolabeling in the DG was clearly different in all pilocarpine-treated animals at all time intervals examined (Fig. 1c–j′) compared to that observed in control rats (Fig. 1a–b′). The main differences were observed in the IML and SGL and involved both diffuse and large VGLUT2-containing terminal labeling. Furthermore, these labeling patterns for VGLUT2 clearly evolved following pilocarpine injection (Fig. 1c–j′). Thus, at 1 week after pilocarpine injection (Fig. 1c–d′), differences in the labeling patterns for VGLUT2 were principally observed in the dorsal DG (Fig. 1c, c′). A loss of diffuse VGLUT2 immunolabeling was evident in the IML of the dorsal DG at both rostral (Fig. 1c, c′) and caudal level (Fig. 1d). This loss contrasted with an apparent increased labeling for large VGLUT2-containing terminals in the SGL of the dorsal DG (Fig. 1c′, arrows) compared with control animals (Fig. 1a′). Two weeks after pilocarpine injection (Fig. 1e–f′), the loss of the diffuse VGLUT2 immunolabeling in the IML was still evident in the dorsal DG at both rostral (Fig. 1e, e′) and caudal (Fig. 1f) levels. Furthermore, the labeling pattern for large VGLUT2-containing terminals (arrows), in 2-week pilocarpine-treated animals, significantly differed from that observed in control but also in 1-week pilocarpine-treated rats. In addition to the numerous VGLUT2-containing large boutons present in the SGL of the DG, many large terminals were now observed in the IML. This aberrant distribution pattern of large VGLUT2-containing terminals in the IML was particularly obvious in the dorsal DG (Fig. 1e′) and contrasted with the restricted localization of these terminals in the SGL in control (Fig. 1a′) and 1-week pilocarpine-treated (Fig. 1c′) rats. An apparent increase of the large VGLUT2-containing boutons was also observed in the entire IML of the ventral DG in 2-week pilocarpine-treated animals (Fig. 1f′, arrows) compared to control (Fig. 1b′) and 1-week pilocarpine-treated (Fig. 1d′) rats. All epileptic animals at 2 (Fig. 1g–h′) and 4 (not shown) months after pilocarpine injection showed similar labeling patterns for VGLUT2. These VGLUT2 labeling patterns displayed marked differences with those from control (Fig. 1a–b′) but also pilocarpine-treated animals at early (1 week; Fig. 1c–d′) and late (2 weeks; Fig. 1e–f′) stages of the latent period. The loss of diffuse labeling observed in IML of the dorsal DG at 1 and 2 weeks was no longer observed at this stage (compare Fig. 1g′ with Fig. 1c′, e′). Furthermore, in these epileptic animals, numerous large terminals labeled for VGLUT2 (arrows) invaded the entire IML throughout all the rostro-caudal extent of the DG (Fig. 1g–h′). This increased labeling of large VGLUT2-containing terminals in the IML, compared with control, 1-week but also 2-week pilocarpine-treated animals, was particularly prominent in the dorsal DG (compare Fig. 1g′ with Fig. 1a′, c′, e′), but was also evident in the ventral DG (compare Fig. 1h′ with Fig. 1b′, d′, f′).

Interestingly, this dynamic reorganization of VGLUT2 immunolabeling observed during the latent period continued to evolve during the chronic stage. Epileptic rats experiencing spontaneous seizures for several months (pilocarpine-treated animals at 12 months) displayed an aberrant labeling pattern for VGLUT2 in the IML of the DG at all rostro-caudal levels examined (Fig. 1i–j), which differed significantly from that of same-age control rats and pilocarpine-treated rats during the latent but also from that of epileptic animals at 2–4 months. Twelve-month pilocarpine-treated rats displayed main differences compared to pilocarpine-treated animals at 2–4 months. These included a complete loss of diffuse VGLUT2 immunolabeling in the IML throughout the entire rostro-caudal extent of the DG (Fig. 1i–j′) and a double-band distribution pattern for large VGLUT2-containing terminals (arrows), predominantly observed in the dorsal DG at rostral level (Fig. 1i, i′). Indeed, at this level, the large VGLUT2-containing terminals were mainly located in the SGL and in the uppermost part of the inner one-third molecular layer (Fig. 1i′, arrows). At caudal level (Fig. 1j, j′), VGLUT2-containing terminals were distributed in the entire IML, but mainly concentrated in the upper part of the inner one-third molecular layer along the dorsal (Fig. 1j)–ventral (Fig. 1j, j′, arrows) extent of the DG.

These aberrant VGLUT2-immunolabeling patterns in pilocarpine-treated animals suggested a marked reorganization of DG afferents originating from SuM neurons.

### Aberrant distribution of fibers and axon terminals originating from SuM neurons in the DG of pilocarpine-treated rats

To further test the above hypothesis, we investigated the labeling pattern of supramammillary–dentate gyrus pathways in epileptic pilocarpine-treated rats at 2–4 months and control animals using BDA anterograde tracer injected in the SuM region. The labeling pattern of BDA-containing fibers and axon terminals was examined in the DG of control (*n* = 3) and epileptic pilocarpine animals (*n* = 6) in which the BDA injection site included neurons from both SuML and SuMM regions.

**Fig. 2:**
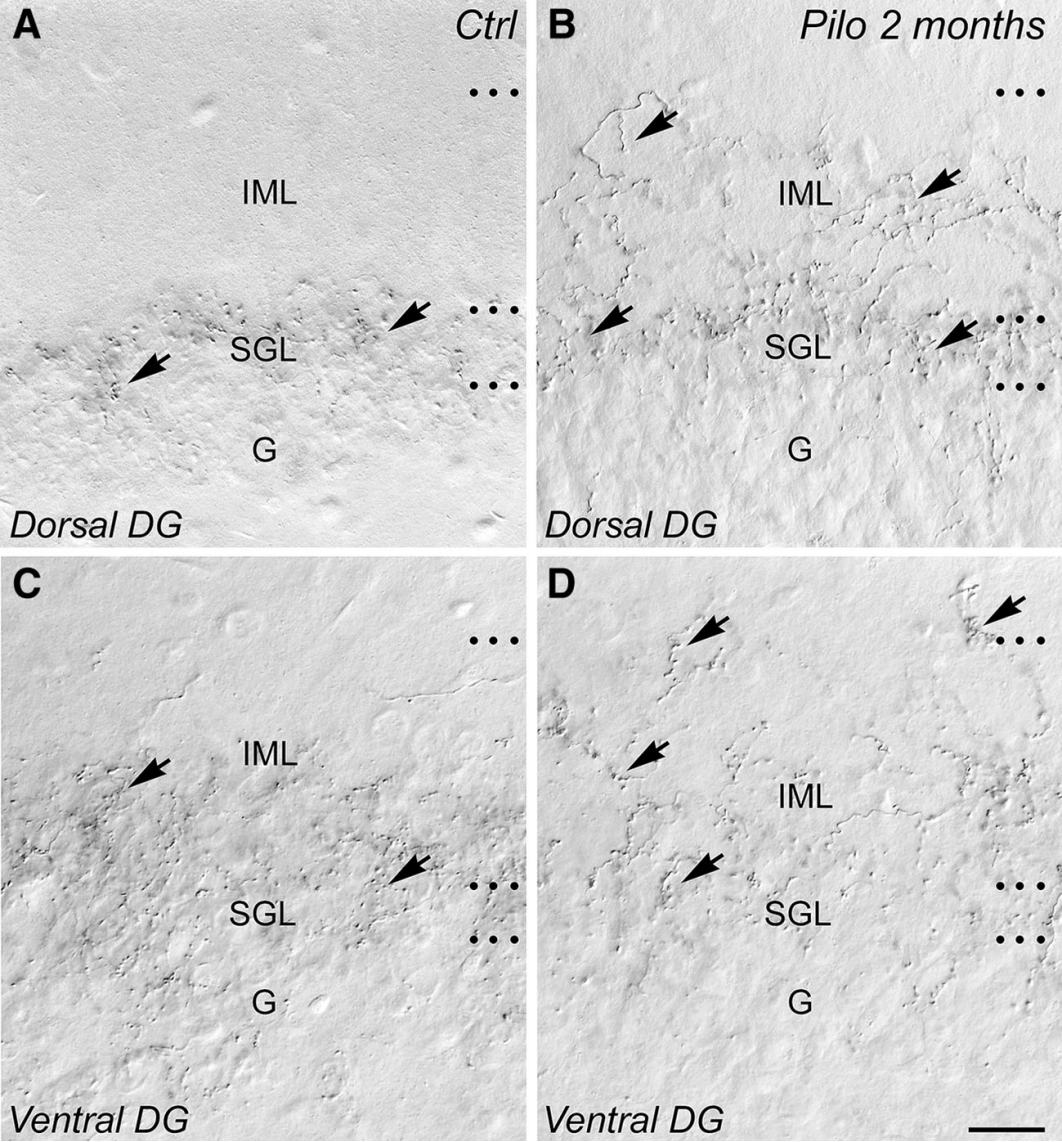
Comparison of the supramammillary–dentate gyrus pathways in coronal sections of dorsal (**a**, **b**) and ventral (**c**, **d**) dentate gyrus from control (**a**, **c**) and epileptic pilocarpine-treated animals (**b**, **d**) revealed with BDA anterograde tracing. **a**–**d** In control as in epileptic animals, the detection of BDA anterograde tracer was performed 10 days after its injection in a region including both the lateral and medial parts of the supramammillary nucleus (SuM). **a**, **c** In a control rat, BDA-containing fibers and axon terminals (*arrows*) were restricted to the supragranular layer (SGL) in dorsal DG (**a**), whereas they were more distributed within the IML in the ventral DG (**c**). **b**, **d** In an epileptic animal at 2 months after pilocarpine injection, an aberrant distribution of BDA-containing fibers and axon terminals was evident. In the dorsal DG (**b**), these fibers and axon terminals (*arrows*) were located not only in the SGL as in a control rat but also invaded the entire IML (compare with panel **a**). In the ventral DG (**d**), many BDA-containing fibers and axon terminals (*arrows*) were also observed in the uppermost part of the IML in contrast to control animal (compare with panel **c**). *DG* dentate gyrus, *G* granule cell layer, *SGL* supragranular layer, *IML* inner molecular layer, *Ctrl* control, *Pilo 2* *months* pilocarpine-treated rat at 2 months after status epilepticus. *Scale bar* 20 µm

In control rats, many anterograde-labeled fibers and axon terminals were observed in the dorsal (Fig. 2a, arrows) and ventral (Fig. 2c, arrows) DG ipsilateral to the injection site and to a lesser extent in the contralateral DG (not shown). BDA-labeled fibers and terminals were restricted to the SGL in the dorsal DG (Fig. 2a, arrows), whereas they were slightly more dispersed within the SGL and IML in the ventral DG (Fig. 2c, arrows). These data are in keeping with previously described results (Haglund et al. 1984; Vertes 1992; Maglóczky et al. 1994; Soussi et al. 2010).

Epileptic pilocarpine-treated animals displayed an aberrant distribution pattern for BDA-containing fibers and axon terminals compared to control rats. This was particularly evident in the dorsal DG in which relatively thick-labeled fibers were giving rise to many thinner axonal branches with numerous en passant boutons not only in the SGL but throughout the entire IML (Compare Fig. 2b to Fig. 2a). An aberrant distribution of BDA-containing fibers and axon terminals was also observed in the ventral DG of chronic pilocarpine-treated rats. Indeed, many fibers and axon terminals were present in the uppermost part of the IML in the ventral DG of chronic pilocarpine-treated but not of control rats (compare Fig. 2d to Fig. 2c).

These results demonstrated a reorganization of the SuM–DG pathways, which displayed a distribution pattern in keeping with the reorganization of the large VGLUT2-containing boutons within the dentate IML of epileptic pilocarpine-treated animals.

**Fig. 3:**
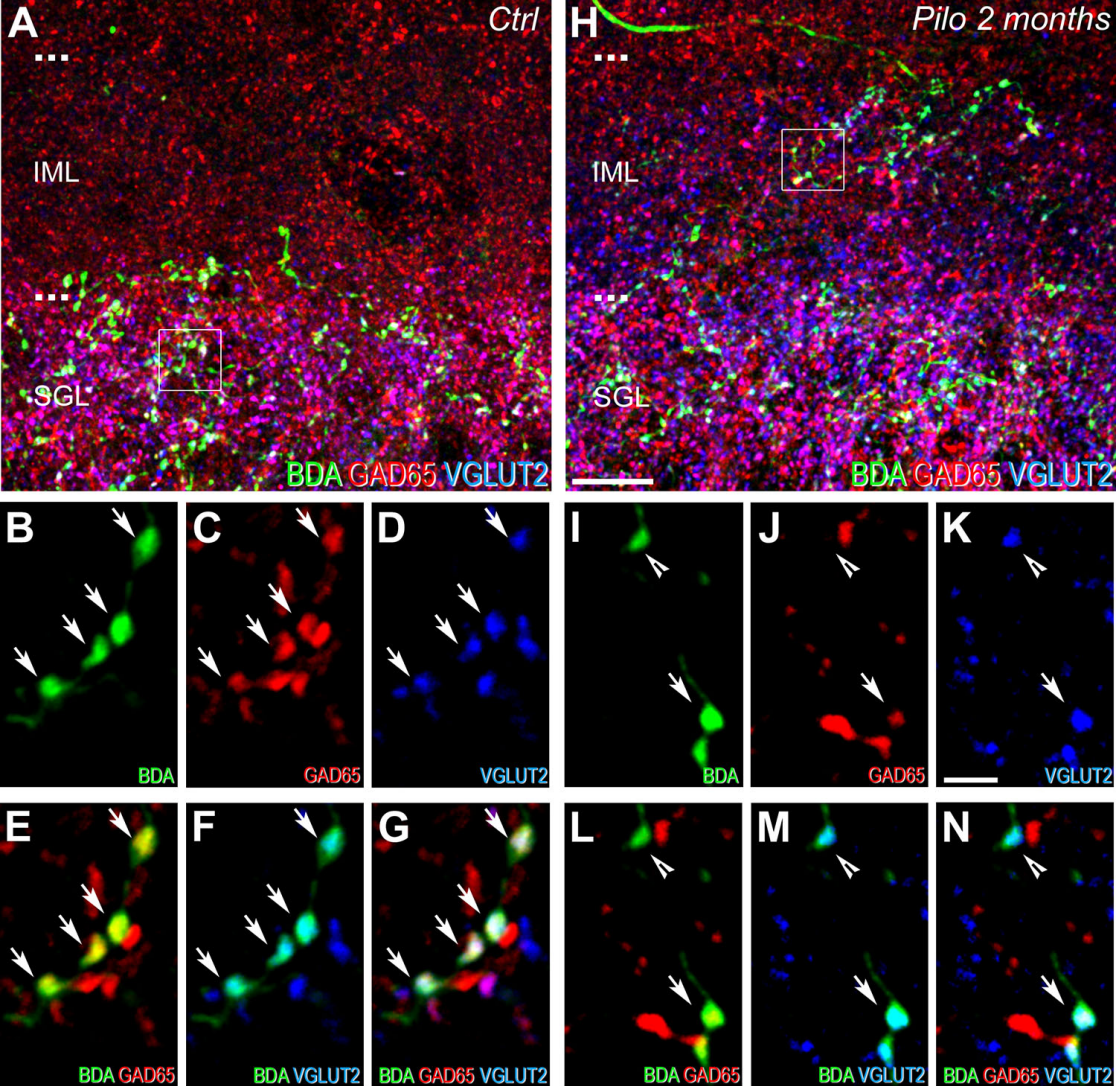
Comparison of neurochemical phenotypes for the dorsal dentate gyrus afferents from the SuM between control (**a**–**g**) and epileptic pilocarpine-treated (**h**–**n**) rats, characterized by simultaneous labeling for BDA anterograde tracer (*green*), GAD65 (*red*) and VGLUT2 (*blue*) in coronal sections. **a** Image corresponding to a maximum intensity projection of a confocal slice z-stack (22 optical slices, spaced at 285 nm) showing labeling for BDA (*green*), GAD65 (*red*) and VGLUT2 (*blue*) in the dorsal DG of a control rat. Axon terminals and fibers, originating from SuM neurons and labeled for the BDA anterograde tracer (*green*), were located mainly in the SGL. Numerous GAD65-containing terminals (*red*) were present in the IML and SGL. VGLUT2-containing terminals (*blue*) were mainly located in the SGL. **b**–**d** Images of the three different fluorophores used for the triple labeling, obtained by sequential acquisition of separate wavelength channels from a single confocal slice in the SGL of the dorsal DG demonstrated that many if not all axon terminals labeled for BDA (**b**, *green*, *arrows*) contained GAD65 (**c**, *red*, *arrows*) and VGLUT2 (**d**, *blue*, *arrows*). **e** Merge of **b** and **c**. **f** Merge of **b** and **d**. **g** Merge of **b**–**d**. Triple-labeled boutons for BDA, GAD65 and VGLUT2 (*white*, *arrows*) were surrounded by double-labeled terminals for GAD65 and VGLUT2 (*purple*) as well as single-labeled terminals for GAD65 (*red*) or VGLUT2 (*blue*). **h** Image corresponding to a maximum intensity projection of a confocal slice z-stack (22 optical slices, spaced at 285 nm) showing labeling for BDA (*green*), GAD65 (*red*) and VGLUT2 (*blue*) in the dorsal DG of an epileptic rat at 2 months after pilocarpine injection. Axon terminals and fibers, originating from SuM neurons, labeled for the BDA anterograde tracer (*green*) were distributed within the entire IML in contrast to the control rat (compare with panel **a**). Numerous GAD65-containing terminals (*red*) were present in the IML and SGL. VGLUT2-containing terminals (*blue*) were located in the SGL but also in all the IML. **i**–**k** Images of the three different fluorophores used for the triple labeling, obtained by sequential acquisition of separate wavelength channels from a single confocal slice, in the IML of the dorsal DG demonstrated two types of BDA-labeled axon terminals (**i**, *green*): the first one contained GAD65 (**j**, *red*, *arrow*) and VGLUT2 (**k**, *blue*, *arrow*), the second one contained VGLUT2 only (**i**–**k**, *arrowhead*). **l** Merge of **i** and **j**. **m** Merge of **i** and **k**. **n** Merge of **i**–**k**. Triple-labeled boutons for BDA, GAD65 and VGLUT2 (*white*, *arrow*) were surrounded by double-labeled terminals for BDA and VGLUT2 (*arrowhead*) as well as single-labeled terminals for GAD65 (*red*) or VGLUT2 (*blue*). *Ctrl* control, *Pilo 2 months* pilocarpine-treated rat at 2 months after status epilepticus. *Scale bars* 10 µm in **a** and **h**; 2 µm in **b**–**g** and **i**–**n**

**Fig. 4:**
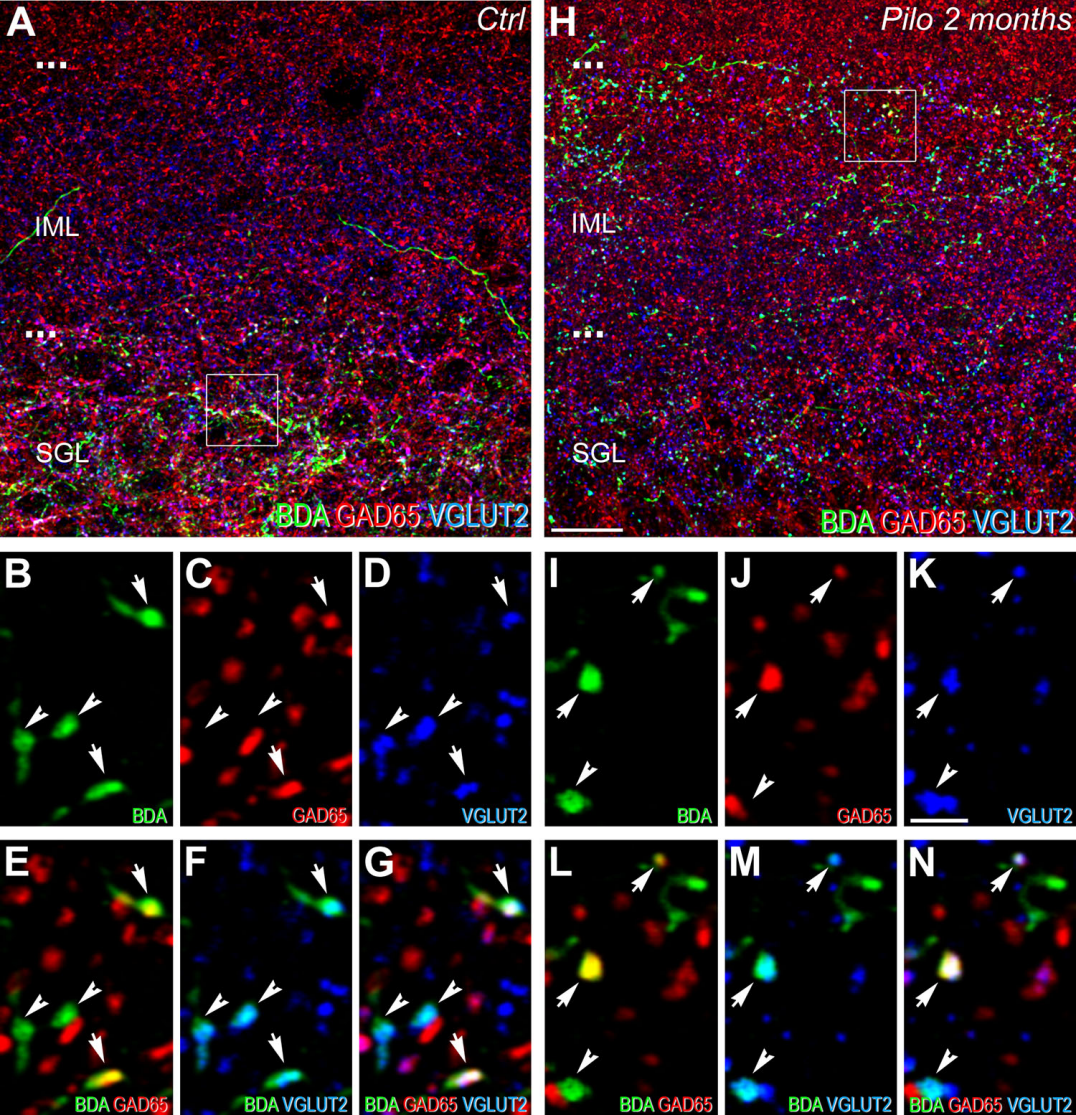
Comparison of neurochemical phenotypes for the ventral dentate gyrus afferents from the SuM between control (**a**–**g**) and epileptic pilocarpine-treated (**h**–**n**) rats, characterized by simultaneous labeling for BDA anterograde tracer (*green*), GAD65 (*red*) and VGLUT2 (*blue*) in coronal sections. **a** Image corresponding to a maximum intensity projection of a confocal slice z-stack (40 optical slices, spaced at 300 nm) showing labeling for BDA (*green*), GAD65 (*red*) and VGLUT2 (*blue*) in the ventral DG of a control rat. Axon terminals and fibers, originating from the SuM neurons, labeled for the BDA anterograde tracer (*green*) were distributed in the SGL and in the lower part of the IML. Numerous GAD65-containing terminals and VGLUT2-containing terminals were present in this region. **b**–**d** Images of the three different fluorophores used for the triple labeling, obtained by sequential acquisition of separate wavelength channels from a single confocal slice, in the SGL of the ventral DG demonstrated axon terminals labeled for BDA (**b**, *green*, *arrows*) containing GAD65 (**c**, *red*, *arrows*) and VGLUT2 (**d**, *blue*, *arrows*) and BDA-labeled terminals containing VGLUT2 only (**d**, *blue*, *arrowheads*). **e** Merge of **b** and **c**. **f** Merge of **b** and **d**. **g** Merge of **b**–**d**. Triple-labeled boutons for BDA, GAD65 and VGLUT2 (*white*, *arrows*) and double-labeled boutons for BDA and VGLUT2 (*arrowheads*) were surrounded by double-labeled terminals for GAD65 and VGLUT2 (*purple*) as well as single-labeled terminals for GAD65 (*red*) or VGLUT2 (*blue*). **h** Image corresponding to a maximum intensity projection of a confocal slice z-stack (40 optical slices, spaced at 300 nm) showing labeling for BDA (*green*), GAD65 (*red*) and VGLUT2 (*blue*) in the ventral DG of an epileptic rat at 2 months after pilocarpine injection. Axon terminals and fibers, originating from SuM neurons, labeled for the BDA anterograde tracer (*green*) displayed an aberrant distribution as compared to the control rat (**a**), many boutons and fibers being observed in the entire IML including the upper part. Numerous GAD65-containing terminals (*red*) and VGLUT2-containing terminals (*blue*) were also present in this entire region. **i**–**k** Images of the three different fluorophores used for the triple labeling, obtained by sequential acquisition of separate wavelength channels from a single confocal slice, in the IML of the ventral DG demonstrated that many of these ectopic BDA-labeled axon terminals (**i**, *green*, *arrows*) contained GAD65 (**j**, *red*, *arrows*) and VGLUT2 (**k**, *blue*, *arrows*) and some contained VGLUT2 only (**i**–**k** arrowhead). **l** Merge of **i** and **j**. **m** Merge of **i** and **k**. **n** Merge of **i**–**k**. Triple-labeled boutons for BDA, GAD65 and VGLUT2 (*white*, *arrows*) and double-labeled terminals for BDA and VGLUT2 (*arrowhead*) were surrounded by double-labeled terminals for GAD65 and VGLUT2 (*purple)* as well as single-labeled terminals for GAD65 (*red)*. *Ctrl* control, *Pilo 2* *months* pilocarpine-treated rat at 2 months after status epilepticus. *Scale bars* 10 µm in **a** and **h**; 2 µm in **b**–**g** and **i**–**n**

Previous studies demonstrated in control rats the heterogeneity of SuM–DG pathways with two main projections originating from neurons in the SuML and the SuMM (Haglund et al. 1984; Vertes 1992; Maglóczky et al. 1994; Vertes and McKenna 2000) that can be identified based on their neurochemical content (Soussi et al. 2010). The axon terminals from the SuML that innervate the SGL of the dorsal DG and to a much lesser extent of the ventral DG contain markers of both glutamatergic (VGLUT2) and GABAergic (GAD65 and VGAT) neurotransmissions, whereas the axon terminals from the SuMM that innervate exclusively the IML contain VGLUT2 only. To assess the contribution of each pathway in the reorganization of the SuM–DG projections, triple immunofluorescence labeling for BDA, VGLUT2 and GAD65 were performed in the dorsal (Fig. 3) and ventral (Fig. 4) regions of the DG in epileptic and control rats.

In the dorsal DG of control rats, virtually all BDA-containing terminals were located in the SGL (Fig. 3a) and were labeled for GAD65 and VGLUT2 (Fig. 3a, g, arrows), in keeping with previously reported data (Boulland et al. 2009; Soussi et al. 2010). The dorsal DG of chronic pilocarpine-treated animals displayed aberrant distribution of BDA-containing terminals not only from an anatomical but also a neurochemical point of view. In the dorsal DG of epileptic rats, BDA-containing terminals were present in the IML, in addition to the SGL (Fig. 3h) in contrast to control rats (Fig. 3a). Many of these BDA-containing terminals were labeled for both GAD65 and VGLUT2 (Fig. 3i–n, arrows) and some for VGLUT2 only (Fig. 3i–n, arrowheads).

In the ventral DG of control animals, BDA-containing terminals were distributed in the SGL and lower part of the IML (Fig. 4a). These terminals included some boutons which co-expressed VGLUT2 and GAD65 (Fig. 4b–g, arrows) and boutons labeled for VGLUT2 only (Fig. 4b–g, arrowheads). The ventral DG of chronic pilocarpine-treated rats displayed a clear aberrant anatomical and neurochemical labeling pattern for BDA-containing terminals (Fig. 4h) as compared to that of control rats. This aberrant pattern consisted mainly in the presence of numerous BDA-containing boutons located in the uppermost part of the IML. Most of these boutons co-expressed GAD65 and VGLUT2 (Fig. 4i–n, arrows), whereas some were only labeled for VGLUT2 (Fig. 4i–n, arrowhead). Furthermore, many BDA-containing terminals labeled for VGLUT2 as well as VGLUT2-containing boutons were evident throughout the entire IML of the ventral DG in epileptic animals (Fig. 4h).

Altogether, these results demonstrated an aberrant distribution of fibers and axon terminals originating from neurons of the SuML but also SuMM in epileptic pilocarpine-treated rats.

### Quantitative analysis of the changes in VGLUT2 and colocalized VGAT and VGLUT2 axon terminal labeling levels in epileptic animals

**Fig. 5:**
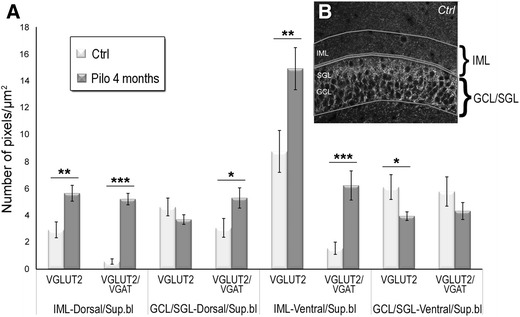
Quantitative analysis of VGLUT2 and VGAT proteins. **a**, **b** Quantitative analysis of the mean densities of labeling for VGLUT2 only and for VGLUT2/VGAT performed for the dorsal and ventral DG, in two regions of interest drawn over the inner molecular layer (IML) and granule cell layer which included the supragranular layer (GCL/SGL) as illustrated in (**b**) of the suprapyramidal blade (Sup.bl). Measures were obtained from three controls (white rectangles) and three pilocarpine-treated rats at 4 months (*gray rectangles*). Statistically significant differences are indicated (*p < 0.05; **p < 0.01; ***p < 0.001; ANOVA test). *Errors bars* SEM

To estimate whether the reorganization of DG afferents originating from the SuML and SuMM corresponded to a dispersion and/or an increased number of boutons, a quantitative analysis of VGLUT2/VGAT- and of VGLUT2-labeled boutons were performed in the entire granule cells layer (GCL) including the supragranular layer (SGL) and in the IML of the dorsal and ventral DG in 4-month pilocarpine-treated (*n* = 3) and control rats (*n* = 3). Quantitative data (Fig. 5a) showed that, in epileptic rats, the average densities (number of pixel/µm^2^) of VGLUT2/VGAT labeling were significantly increased, compared to control animals, in the IML (+814 %; Pilo: 5.21 ± 0.43; Ctrl: 0.57 ± 0.2; *p* < 0.001) and GCL/SGL (+73 %; Pilo: 5.30 ± 0.75; Ctrl: 3.07 ± 0.7; *p* < 0.01) of the dorsal DG as well as in the IML (+299 %; Pilo: 6.22 ± 1.08; Crtl: 1.56 ± 0.48; *p* < 0.05) of the ventral DG. No difference was found in the GCL/SGL of the ventral DG (Pilo: 4.33 ± 0.63; Ctrl: 5.78 ± 1.09). The average densities of VGLUT2 labeling were significantly increased in the IML of the dorsal (+93 %; Pilo: 5.65 ± 0.59; Ctrl: 2.93 ± 0.59; *p* < 0.01) and ventral (+70 %; Pilo: 14.91 ± 1.57; Ctrl: 8.75 ± 1.56, *p* < 0.01) DG and significantly decreased in the GCL/SGL of the ventral DG (−35 %; Pilo: 3.94 ± 0.32; Ctrl: 6.11 ± 0.92; *p* < 0.05) of epileptic compared to control animals. The average densities of VGLUT2 labeling were not significantly different in the GCL/SGL of the dorsal DG between epileptic (3.69 ± 0.35) and control (4.63 ± 0.66) rats.

These results demonstrated an overall increased density of labeling for terminals displaying SuML (VGLUT2/VGAT) as well as SuMM (VGLUT2) neurochemical phenotypes within the dentate IML of chronic pilocarpine-treated animals.

### Granule cells did not express VGLUT2 in control and pilocarpine-treated animals

**Fig. 6:**
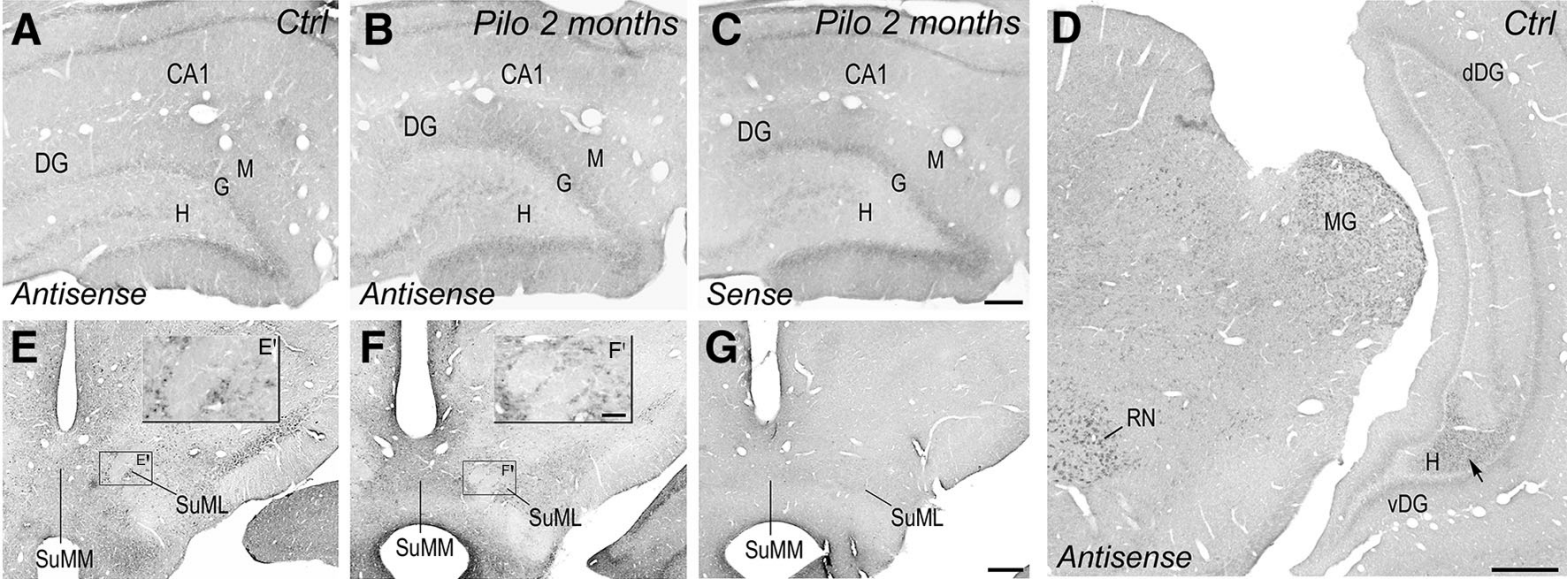
Comparison of labeling for VGLUT2 mRNA in coronal sections of hippocampal formation (**a**–**d**) and of the SuM (**e**–**g**) from control (**a**, **d**, **e**) and epileptic pilocarpine-treated rats (**b**, **c**, **f**, **g**), processed for the same color reaction time. Sections of the hippocampal formation from a control rat (**a**) and an epileptic rat at 2 months (**b**), processed with antisense RNA probe, showed a faint nonspecific labeling for VGLUT2 in the pyramidal and granule cell layers similar to that observed in a section from an epileptic rat processed for the control sense RNA probe (**c**). **d** A section of the hippocampal formation, at a caudal level in a control rat, showing that hilar neurons, presumed mossy cells, in the ventral DG (vDG) expressed low levels of VGLUT2 mRNA as compared to mesencephalic nuclei including the red nucleus (RN) and the medial geniculate (MG). Hilar neurons in the dorsal DG (dDG) did not display detectable level of VGLUT2 mRNA. **e** In a section from a control rat, processed with antisense RNA probe, VGLUT2 mRNA was expressed by many diencephalic neurons including neurons located in the medial part (SuMM) and lateral part (SuML) of the supramammillary nucleus (SuM) as illustrated at higher magnification in the *inset* (**e**′). **f** In a section from an epileptic rat at 2 months after pilocarpine injection, processed with antisense RNA probe, revealed similar pattern and intensity of labeling for VGLUT2 mRNA in the SuMM and SuML (see *inset* **f**′) as that observed in the control rat (compare with **e**, **e**′). **g** No labeling was observed in an adjacent section from an epileptic rat at 2 months after pilocarpine injection, processed with sense RNA probe. *Ctrl* control, *Pilo 2* *months* pilocarpine-treated rat at 2 months after status epilepticus. *Scale bars* 100 µm in **e** **f**, **g**; 25 µm in **e**′, **f**′; 50 µm in **a**–**c**; 500 µm in **d**

Sprouting of mossy fibers, the axons of dentate granule cells, in the IML of pilocarpine-treated rats is well established (Okazaki et al. 1995; Okazaki and Nadler 2001; Buckmaster et al. 2002). Even though in adult rodent brain, VGLUT1 appears to be the only vesicular glutamate transporter expressed by dentate granule cells (Fremeau et al. 2001; Herzog et al. 2001; Kaneko et al. 2002; Fremeau et al. 2004; Herzog et al. 2006), a transient VGLUT2 expression by granule cells at early postnatal developmental stages, that rapidly decreases with age, was reported in mouse (Fremeau et al. 2004; Herzog et al. 2006). We therefore investigated whether the aberrant pattern of VGLUT2 labeling in the IML could partly reflect an ectopic expression of VGLUT2 by dentate granule cells in pilocarpine-treated rats. Nonradioactive in situ hybridization experiments for the detection of VGLUT2 mRNA with antisense probes demonstrated very low levels of labeling in all principal layers of the hippocampus including the pyramidal and dentate granule cell layers of control (Fig. 6a) as well as pilocarpine-treated animals (Fig. 6b). These low levels of labeling were similar to that observed with sense probes in pilocarpine-treated (Fig. 6c) or control (not shown) rats and therefore corresponded to nonspecific labeling. Whereas no detectable specific labeling was observed in the dorsal DG, hilar neurons in the ventral DG displayed low levels of VGLUT2 mRNA (Fig. 6d). This low level of mRNA in the hilar neurons of the ventral DG contrasted with the moderate to high levels of VGLUT2 mRNA labeling observed in the SuML and SuMM of pilocarpine-treated animals (Fig. 6f, f′) and control rats (Fig. 6e, e′). In both animal groups, VGLUT2 mRNA-expressing neurons within the SuM displayed comparable levels of labeling, using the antisense probes, whereas no labeling was observed with the sense control probes (Fig. 6g).

These results demonstrated that dentate granule cells did not express VGLUT2 mRNA in control as well as in epileptic pilocarpine-treated rats.

### Aberrant distribution of VGLUT2 immunolabeling is not related with granule cell dispersion or bi-lamination

Granule cells dispersion and bi-lamination have been described in humans with mesial temporal lobe epilepsy as well as in experimental animal models (Houser 1990; Mello et al. 1993; Haas et al. 2002; Chai et al. 2013). We therefore investigated whether the aberrant distribution of VGLUT2-containing axon terminals in the dentate IML of pilocarpine-treated animals including the double-band distribution observed in 12-month epileptic animals could result from a dispersion or a bi-lamination of granule cells.

**Fig. 7:**
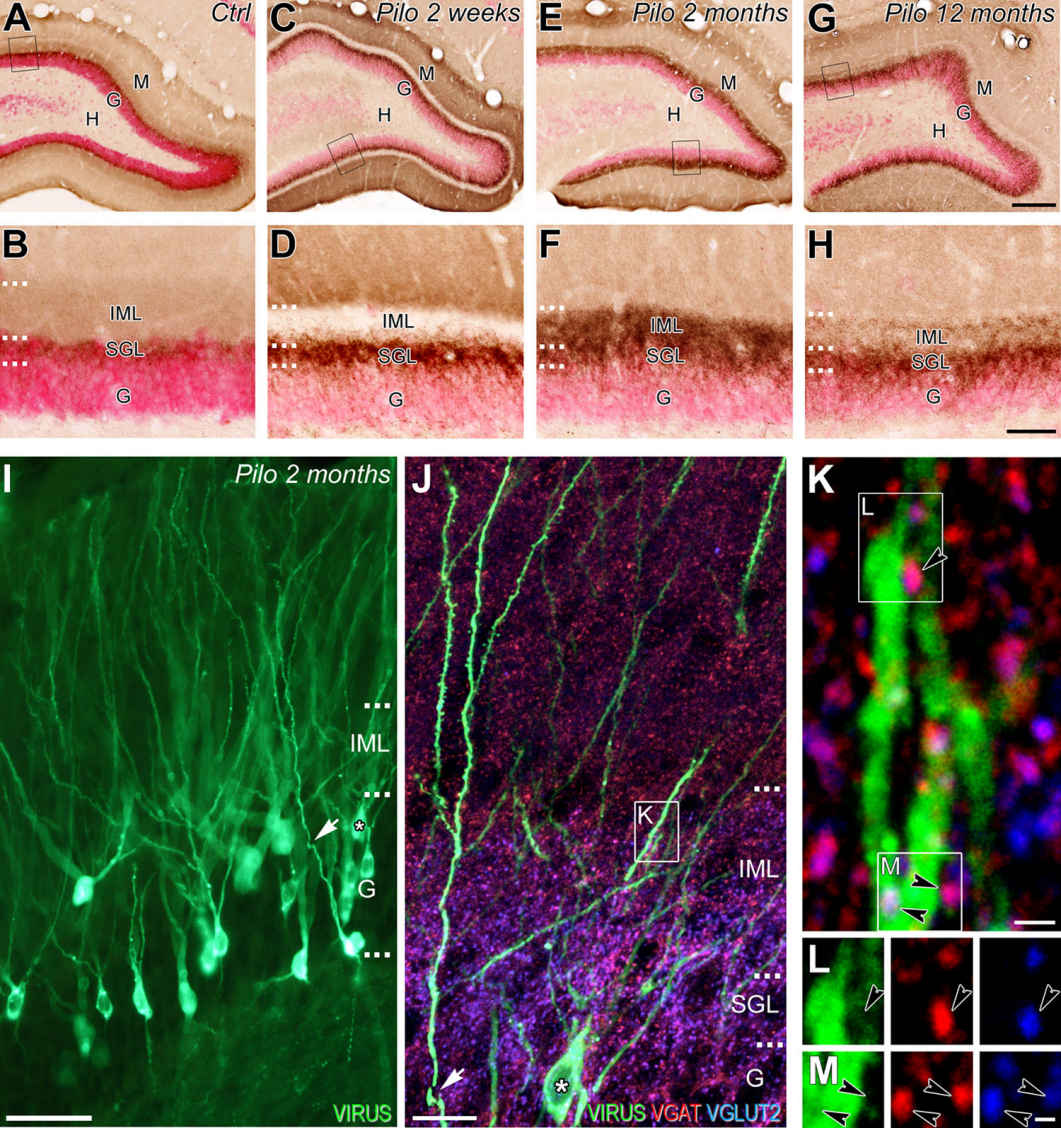
Synaptic targets of ectopic dentate gyrus afferents from SuM neurons in pilocarpine-treated rats. **a**–**h** Comparison of distribution patterns of VGLUT2-containing terminals (*brown*) and NeuN-labeled dentate granule cells (*red*) in coronal sections of the dorsal DG from a control rat (**a**, **b**) and pilocarpine-treated animals at 2 weeks (**c**, **d**), 2 months (**e**, **f**) and 12 months (**g**, **h**). **b**, **d**, **f**, **h** High magnifications of outlined areas, respectively, illustrated in panels **a**, **c**, **e** and **g**. In pilocarpine-treated rats (**c**–**h**), the distribution pattern of dentate granule cell somata was similar to that observed in the control animal (**a**, **b**). No dispersion or bi-lamination of granule cells was associated with the aberrant VGLUT2-immunolabeling observed in the IML in pilocarpine-treated rat from 2 weeks on. **i** A section of dorsal DG from an epileptic animal at 2 months that was injected into the CA3 stratum lucidum of the dorsal hippocampus with the Rabies virus (RV) retrograde tracer to label cell bodies and dendritic trees of dentate granule cells (G). **j**–**k** Image corresponding to a maximum intensity projection of a confocal slice z-stack (20 optical slices, spaced at 296 nm) showing labeling for the RV (*green*), VGAT (*red*) and VGLUT2 (*blue*) in the dorsal dentate gyrus of an epileptic rat at 2 month after pilocarpine injection. **j** High magnification of the region indicated by an arrow and a star in panel (**i**). Presumed axon terminals from SuM neurons, labeled for VGAT and VGLUT2, displayed an aberrant distribution with many boutons present in the entire IML including the upper part. Note that in the epileptic rat, the proximal apical dendrites (*arrow*) of dentate granule cells (*) across the IML displayed a low number of spines as compared to more distal segment. **k** Higher magnification of the outlined region indicated in panel **j** showing a dendritic tree of dentate granule cell across the upper IML contacted by ectopic VGLUT2/VGAT-containing axon terminals. **l**, **m** Images of the three different fluorophores used for the triple labeling, obtained by sequential acquisitions of separate wavelength channels from a single confocal slice, in the two outlined regions of the IML indicated in panel **k** and demonstrating that ectopic axon terminals labeled for VGAT (*red*, *arrowhead*) and VGLUT2 (*blue*, *arrowhead*), presumably originating from the SuM establish contact on the dendritic shafts of dentate granule cells retrogradely labeled with rabies virus (*green*). *Scale bars* 200 µm in **a**, **c**, **e** and **g**; 50 µm in **b**, **d**, **f** and **h**; 100 µm in **i**; 15 µm in **j**; 1 µm in **k**; 0.5 µm in **l**, **m**

The distribution pattern of VGLUT2 immunolabeling associated with that of NeuN-labeled dentate granule cells was analyzed in control (Fig. 7a, b) and pilocarpine-treated animals at 2 weeks (Fig. 7c, d), 2 months (Fig. 7e, f) and 12 months (Fig. 7g, h). No dispersion or bi-laminar organization of dentate granule cells correlated with the aberrant pattern of VGLUT2-containing terminals in the IML of all pilocarpine-treated rats examined (Fig. 7c–h). The thickness of the dentate granule cell layer was relatively similar in control and pilocarpine-treated rats, even though dentate granule cells in pilocarpine-treated animals displayed a decreased labeling intensity for NeuN (Fig. 7c–h) compared to control rats (Fig. 7a, b). Virtually no NeuN-labeled cell body was evident in the IML of pilocarpine-treated animals displaying increased number of VGLUT2-containing terminals at 2 weeks (Fig. 7c, d), 2 months (Fig. 7e, f) and 12 months (Fig. 7g, h) after pilocarpine injection.

### Synaptic targets of ectopic VGLUT2/VGAT- and VGLUT2-containing boutons within the IML of epileptic rats

The synaptic targets of aberrant VGLUT2/VGAT- and VGLUT2-containing axon terminals in the dentate IML of pilocarpine-treated animals were investigated. Retrograde labeling with Rabies virus resulting in a Golgi-like labeling of the dendritic arbor of dentate granule cells (Fig. 7i) revealed that in epileptic pilocarpine-treated rats, the proximal dendrites of these cells across the IML displayed a few number of spines as compared to more distal segment (Fig. 7j). Along the IML, these spine-free dendritic trees were contacted by many ectopic VGLUT2/VGAT- and VGLUT2-containing axon terminals (Fig. 7k, l, m, arrows).

## Discussion

This study demonstrates a marked reorganization of DG afferents from neurons of the SuM in the pilocarpine model of MTLE. This reorganization, characterized by an aberrant distribution and an increased number of fibers and axons terminals from neurons of the SuML and the SuMM within the entire IML of the DG, most likely results from sprouting of these SuM–DG afferents. The aberrant connections formed by SuM neurons within the IML of the DG which start to appear during the latent period and are numerous when animals display spontaneous seizures could contribute along with the reorganization of hippocampal intrinsic networks to the emergence of spontaneous seizures.

### General considerations

Our data demonstrate two patterns for VGLUT2 immunohistochemical labeling in control rats in keeping with previous studies using identical or different antibodies for VGLUT2 detection (Fremeau et al. 2001; Halasy et al. 2004). The first one corresponds to large punctate structures, and the second one to a diffuse and thin labeling. Whereas it is now demonstrated that the large punctate structures, located in the supragranular layer of the DG, correspond for a large majority to axon terminals from SuM neurons (Boulland et al. 2009; Soussi et al. 2010), the specificity and origin of the diffuse labeling is debated (Halasy et al. 2004). However, it was suggested that this diffuse labeling for VGLUT2 observed in the IML of the DG could correspond to the axon terminals of glutamatergic mossy cells (Halasy et al. 2004). Several of our results support this hypothesis. The first one is provided by our in situ hybridization experiments showing that hilar neurons in the ventral DG of control rats express low levels of VGLUT2 mRNA despite no detectable level is evident for hilar neurons of the dorsal DG. The second set of data, supporting this hypothesis, is the loss of the VGLUT2 diffuse thin labeling in the IML of the dorsal DG observed during the latent period at 1 and 2 weeks after pilocarpine-induced status epilepticus. Such loss is well correlated with the death of hilar glutamatergic mossy cells and associated axon terminals in the IML of the dorsal DG, reported in many experimental models of temporal lobe epilepsy (Sloviter 1987; 1991; Lowenstein et al. 1992; Sloviter et al. 2003; Zappone and Sloviter 2004; Kienzler et al. 2009), including the pilocarpine model (Obenaus et al. 1993; Buckmaster et al. 2002; Ferhat et al. 2003; Sloviter et al. 2003; Boulland et al. 2007; Sbai et al. 2012). The transient recovery of this loss of diffuse labeling at 2–4 months is in keeping with partial compensation provided by remaining mossy cells including from the ventral DG. The striking loss of VGLUT2 diffuse labeling observed in the IML of the DG at 12 months after pilocarpine treatment is likely to reflect further degeneration of mossy cells including those from the ventral DG observed in epileptic animals experiencing severe seizures (for review see Kienzler et al. 2009) as well as in patients suffering from mesial temporal lobe epilepsy (Blümcke et al. 2000).

### Reorganization of SuM–DG pathways

Our data demonstrate a marked reorganization of DG extrinsic afferents from the SuM. Such reorganization, which starts during the latent period, is massive at 2–4 months after pilocarpine injection, when animals display frequent spontaneous seizures, and continues to evolve after several months of seizures. This reorganization is illustrated by an aberrant distribution of large VGLUT2-containing terminals in regions that are not innervated in control rats. The SuM origin of these large VGLUT2-containing terminals displaying ectopic distribution within the entire IML of epileptic animals is supported by our following results: (1) An aberrant distribution of anterogradely labeled BDA-containing terminals from SuM neurons demonstrated by the presence of fibers and terminals in the entire IML of epileptic pilocarpine animals instead of being restricted to the SGL as in control rats; (2) Many if not all BDA-containing terminals with ectopic localization display the neurotransmitter phenotype of terminals from SuML neurons which co-express VGLUT2 and GAD65 or VGAT or phenotype from SuMM neurons expressing VGLUT2 only (Boulland et al. 2009; Soussi et al. 2010).

Furthermore, our data strongly suggest that none of the aberrant VGLUT2-containing terminals correspond to the terminals of mossy fibers, the axons of glutamatergic granule cells, known to sprout and form ectopic connections within the IML of the pilocarpine model (Mello et al. 1993; Okazaki et al. 1995; Buckmaster et al. 2002). Indeed, our in situ hybridization experiments do not provide evidence for aberrant expression of VGLUT2 mRNA by granule cells in pilocarpine-treated animals. The levels of labeling in all pilocarpine-treated and control rats obtained with antisense probes were similar to that found with control sense probes. Therefore, we conclude that dentate granule cells do not express VGLUT2 mRNA either in adult control or pilocarpine-treated rats. VGLUT1 appears to be the only vesicular glutamate transporter expressed by granule cells in adult naive (Fremeau et al. 2001; Herzog et al. 2001) and epileptic rats (Boulland et al. 2007).

### Reorganization results from sprouting of axon terminals from SuML and SuMM neurons

Our study provides evidence that in epileptic animals, the aberrant distribution of axon fibers and terminals from SuM neurons innervating the IML of the DG is due to an increased number of axon terminals instead of a dispersion of pre-existing ones. First of all, the emergence of a VGLUT2/VGAT labeling within the IML of the entire DG in these animals, associated with an increase of this labeling within the GCL/SGL, as demonstrated by the quantitative analysis strongly supports an increase of the number of VGLUT2/VGAT-containing boutons originating from the SuML instead of a dispersion of these boutons from the GCL/SGL, in which they are located in control rats (Boulland et al. 2009; Soussi et al. 2010). We cannot completely exclude that part of the increased labeling density observed within the GCL/SGL of the dorsal DG reflects an increase in protein levels within terminals instead of an increased number of boutons within this layer. However, our in situ hybridization experiments do not support high increases of VGLUT2 synthesis in epileptic animals since similar levels of VGLUT2 mRNA was observed in the SuM of control and epileptic rats. Therefore, the massive increase of VGLUT2/VGAT labeling observed within the IML most likely results from newly formed boutons due to a sprouting of axonal branches and terminals from the SuML invading the IML of the entire DG.

Furthermore, our results demonstrate in the epileptic animals a marked increased labeling for VGLUT2 not only in the IML of the ventral but also of the dorsal DG, whereas VGLUT2 labeling was slightly decreased or not modified in the granule cell layer of the ventral and of the dorsal DG, respectively. In naïve rats, axon terminals, containing VGLUT2 only, originate mainly from the SuMM (Soussi et al. 2010), and these afferents from the SuMM innervate the ventral but not the dorsal DG (Maglóczky et al. 1994). Therefore, the marked increased labeling for VGLUT2 in the IML of the entire DG strongly suggests that fibers and axon terminals from the SuMM do not only sprout in the ventral DG but also invade the IML of the dorsal DG. This is further supported by anterograde-tracing experiments showing, within the IML of the dorsal DG in epileptic animals, BDA-containing terminals labeled for VGLUT2 only in addition to many terminals co-labeled for VGLUT2 and GAD65 (originating from the SuML).

Our experiments cannot exclude completely that additional sources of origin could contribute to the increased number of VGLUT2-containing terminals in the DG of epileptic rats. However, in naïve rat, the SuM is the major source for VGLUT2-containing large terminals innervating the DG. Indeed, despite the suggestion that VGLUT2-containing neurons of the medial septum/diagonal band complex (MS/DB) could innervate the DG (Colom et al. 2005), DG afferents from the MS/DB are almost exclusively cholinergic and GABAergic (Nyakas et al. 1987; Freund and Antal 1988; Gaykema et al. 1990). None of these cholinergic or GABAergic neurons contains VGLUT2 (Gritti et al. 2006). Similarly, serotoninergic neurons from the medial raphe innervating the DG (Conrad et al. 1974; Vertes et al. 1999) do not express VGLUT2 mRNA but VGLUT3 (Gras et al. 2002; Herzog et al. 2004). Furthermore, there is no evidence that neurons from the deep layers (IV–VI) of the entorhinal cortex known to project within all molecular layer, including the IML, in the ventral DG (Deller et al. 1996; Deller 1998) express VGLUT2 (Halasy et al. 2004). Finally, neurons of the thalamic nucleus reuniens which express VGLUT2 mRNA (Barroso-Chinea et al. 2007) are known to provide strong innervations to the hippocampal formation; however, their axons form large boutons within the stratum lacunosum of CA1 and not the DG (Wouterlood et al. 1990; Hoover and Vertes 2012). Several cells scattered within various hypothalamic nuclei including the tuberomammillary nucleus and the lateral hypothalamic area have been reported to project to the hippocampal formation (Haglund et al. 1984; Köhler et al. 1984; Köhler et al. 1985; Maglóczky et al. 1994). Whereas these nuclei are likely to innervate the CA2/CA3 regions of the hippocampus, the innervation of the dentate IML as the neurochemical content for VGLUT2 has to be demonstrated. Thus, in the epileptic animals, the increased densities of VGLUT2- and VGLUT2/VGAT-containing boutons invading the IML of the entire DG are likely to be provided mainly by SuMM and SuML neurons.

All together, our data provide strong evidences for an actual sprouting of axon terminals from SuML and SuMM neurons in pilocarpine-induced epileptic animals as previously suggested in human MTLE (Maglóczky et al. 2000) and the related kainate model (Skyers et al. 2003).

### Functional consequences of reorganization of SuM–DG pathways

Our results also demonstrate that the reorganization of SuM–DG including the aberrant distribution and increased densities of fibers and axon terminals from SuML and SuMM neurons within the IML of DG follows the temporal development of epilepsy. It starts during the latent period, becomes significant at the end of this period/beginning of the chronic stage when seizure onset occurs (El-Hassar et al. 2007) and is massive when the frequency of spontaneous seizures reaches a plateau and stabilizes around 2 months after pilocarpine injection (unpublished data), similarly to that described in the kainate-induced seizures model (Williams et al. 2009). This reorganization likely leads to an aberrant connectivity between the SuM and the DG with ectopic and/or newly formed axon terminals targeting different subcellular compartments than in the control rats. In control rats, axon terminals from the SuML innervate the cell bodies and adjacent proximal apical dendrites of dentate granule cells (Maglóczky et al. 1994, 2000; Boulland et al. 2009; Soussi et al. 2010). We show that in epileptic animals, the ectopic axon terminals from the SuML, including those showing a double-band organization, establish presumed synaptic contacts all along dentate granule cell dendrites across the entire IML.

The direct functional consequences of this aberrant connectivity and therefore its contribution to epileptogenesis or ictogenesis are clearly speculative and deserve specific studies that are beyond the scope of this work. However, it is tempting to speculate that such reorganization could facilitate the emergence of spontaneous seizures since the time course of its development follows the emergence and stabilization of the epilepsy. In keeping with this hypothesis, Saji et al. (2000) reported that transient silencing of SuM–hippocampal pathways, performed by injection of muscimol, an agonist of GABA-A receptor into the SuM prevents the genesis and spread of spontaneous seizures in a rat model of kainic acid-induced seizures. Anyway, the physiological consequences of such structural reorganization have to be understood in order to access its potential implication in the epileptic zone.

## Conclusion

Our findings provide evidence for a marked reorganization of both SuM–DG pathways originating from SuML and SuMM neurons in epileptic pilocarpine-treated animals. The reorganization, which reflects axon terminals sprouting, leads to an aberrant connectivity that could play a role in triggering spontaneous seizure according to the emotional and cognitive state of the subject.
